# 635. Do Necrotizing Soft Tissue Infections Behave Differently in Patients with Hematological Malignancies? A Propensity Score-Matched Retrospective Cohort Study in an Oncological Center in Mexico City

**DOI:** 10.1093/ofid/ofae631.200

**Published:** 2025-01-29

**Authors:** Antonio Camiro-Zuñiga, Jack N Salto-Quintana, Edwin Ortega-García, Carolina Pérez-Jiménez, Alexandra Martin-Onraet, Pamela Alatorre Fernandez, Beda Islas-Muñoz, Patricia Cornejo-Juárez, Patricia Volkow-Fernández, Diana Vilar-Compte

**Affiliations:** Instituto Nacional de Cancerologia, Mexico City, Distrito Federal, Mexico; Instituto Nacional de Cancerologia, Mexico City, Distrito Federal, Mexico; Instituto Nacional de Cancerologia, Mexico City, Distrito Federal, Mexico; Instituto Nacional de Cancerologia, Mexico City, Distrito Federal, Mexico; Instituto Nacional de Cancerologia, Mexico City, Distrito Federal, Mexico; Instituto Nacional de Cancerología, Mexico City, Distrito Federal, Mexico; Instituto Nacional de Cancerologia, Mexico City, Distrito Federal, Mexico; Instituto Nacional de Cancerología, Mexico City, Distrito Federal, Mexico; INSTITUTO NACIONAL DE CANCEROLOGÍA, MEXICO CITY, Distrito Federal, Mexico; Instituto Nacional de Cancerología, Mexico City, Distrito Federal, Mexico

## Abstract

**Background:**

Necrotizing Soft Tissue Infections (NSTI) are severe clinical entities that occur in specific clinical contexts. Although, severe neutropenia related to hematological malignancies (HM) has been described in relation with NSTI, there is a paucity of published data describing its course and outcome in this population

**Table 1.**
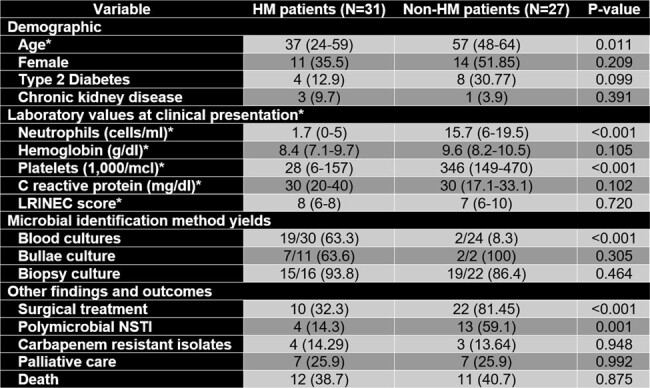
Population characteristics and outcomes All values represented are N(%) unless otherwise noted with an asterisk. Variables marked with an asterisk ( * ), are represented with median(interquartile range).

LRINEC score: Laboratory Risk Indicator for Necrotizing Fasciitis score, it is calculated using values of serum levels of C-reactive protein, White blood cell count, Hemoglobin, Sodium, Creatinine and Glucose.

**Methods:**

We did a retrospective cohort study that included all adults with NSTI treated in the Instituto Nacional de Cancerologia in Mexico City, from 2008 to 2023. We identified all individuals with HM and matched them with individuals with non-hematological cancer using a propensity score based on age, site of infection, and the LRINEC score; and compared their clinical characteristics and outcomes. To determine if severe neutropenia was the main factor driving clinical differences amongst groups, we executed a paired sensitivity analysis comparing three groups: neutropenic adults with HM, non-neutropenic adults with HM, and adults with non-hematological cancer

**Figure 1.**
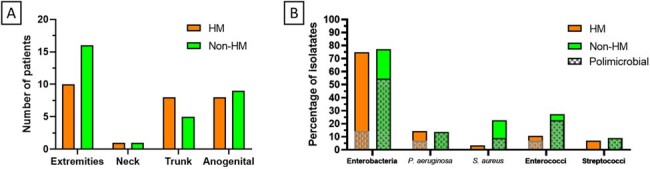
Site of NSTI and identified microbes Panel A: here we show the affected sites in each NSTI per group. There were no significant differences amongst the frequency of the affected sites when comparing both groups. Panel B: here we show the frequency of each category of bacterial isolates, and the frequency in which the isolate presented as part of a polymicrobial infection. Except for S. aureus, there were no significant differences amongst the frequency of the different bacterial isolates when comparing both groups.

**Results:**

We included 31 individuals with HM and NSTI. When compared with the individuals without HM, these were more likely to have severe neutropenia during the NSTI (45.2% vs 3.7%, p< 0.001) and positive blood cultures at NSTI diagnosis (8.3% vs 63.3%, p< 0.001), and less likely to have polymicrobial (59.1% vs 14.3%, p=0.001) or Staphylococcal etiologies (22.7% vs 3.6%, p=0.039). HM patients were less likely to receive surgical treatment (81.5% vs 32.3%, p< 0.001), even though the median time from symptom onset to diagnosis was lower (4 vs 2 days, p=0.009). On the sensitivity analysis, only neutropenic patients had an increased positive yield of blood cultures (p=0.002) and were less likely to receive surgical treatment (p< 0.001). Neutropenic patients showed a higher mortality when compared with non-neutropenic HM patients (63.6% vs 27.3%, p=0.036).

**Figure 2.**
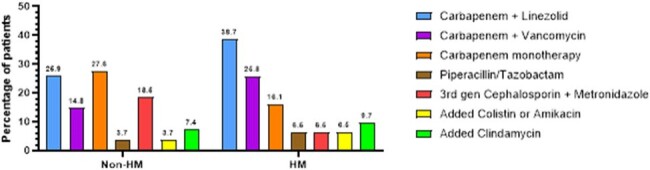
Initial antibiotic regimen

Here we show the different initial antibiotic regimes per group. HM patients had a Carbapenem-based regime more frequently than Non-HM patients (p<0.001), while a regime consisting of a 3rd generation Cephalosporin and Metronidazole was less frequently used in HM patients (p<0.001). There were no significant differences amongst the frequency of additional treatment with Colistin, Amikacin or Clindamycin.

**Conclusion:**

Patients with HM present with bacteriemia and monomicrobial NSTIs due to Gram-negative bacilli, which suggests bacterial translocation as the main etiology of NSTI in this population. Severe neutropenia could be the driving factor causing this phenomenon. Lack of prompt surgical interventions due to associated thrombocytopenia could explain the high mortality of NSTI in this population.

**Table 2.**
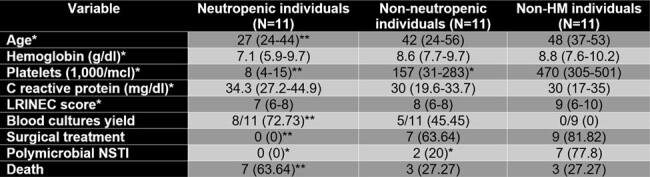
Sensitivity analysis All values represented are N(%) unless otherwise noted with an asterisk. Variables marked with an asterisk ( * ), are represented with median(interquartile range). Neutropenia was defined as having <500 neutrophils/ml. *significant difference when compared with Non-HM users **significant difference when compared with Non-neutropenic users and Non-HM users

**Disclosures:**

**All Authors**: No reported disclosures

